# Very old age impacts masticatory performance: a study among sexagenarians to centenarians

**DOI:** 10.1007/s00784-024-05742-y

**Published:** 2024-06-01

**Authors:** Caroline Sekundo, Cornelia Frese, Niklas Alich, Eva Langowski, Sinclair Awounvo, Diana Wolff, Andreas Zenthöfer

**Affiliations:** 1 Heidelberg University, Department of Conservative Dentistry, University Hospital Heidelberg, Heidelberg, Germany; 2 Institute of Medical Biometry, Heidelberg University, Heidelberg, Germany; 3 Heidelberg University, Department of Prosthodontics, University Hospital Heidelberg, Heidelberg, Germany

**Keywords:** Centenarian, Masticatory performance, Mastication

## Abstract

**Objective:**

This cross-sectional pilot study evaluated the impact of age on masticatory performance among individuals aged 65 to 106 years, as part of the Heidelberg Dental Centenarian Study (HD-100Z) conducted in South-Western Germany.

**Materials and methods:**

A total of 31 centenarians were recruited, alongside 31 individuals each from the age groups 75–99 and 65–74, matched based on sex, prosthetic status, and number of teeth. Masticatory performance was assessed using a two-colored chewing gum test and digital image processing. Multiple linear regression analysis was performed to assess the effect of age, sex, number of teeth, type of prosthesis on the masticatory performance.

**Results:**

Masticatory performance, as measured by the standard deviation of hue in the chewing gum test, decreased significantly in centenarians compared to individuals aged 75–99 years (-0.112, *p* = 0.037) and those aged 65–74 years (-0.274, *p* < 0.001). The effects of sex, number of teeth, and type of prosthesis on masticatory performance were not significant associations (*p* ≥ 0.135).

**Conclusion:**

The findings suggest that age may have a significant influence on masticatory performance in the studied age groups, challenging previous notions that aging itself has little impact on masticatory ability. The inclusion of centenarians in the study highlights the need for further investigation into masticatory function in age groups reaching up to 100 years or more.

**Clinical relevance:**

This study contributes to the understanding of how ageing affects oral function, which may guide dental treatment approaches for older individuals, and set the stage for more in-depth investigations in this field in the future.

**Supplementary Information:**

The online version contains supplementary material available at 10.1007/s00784-024-05742-y.

## Introduction

Mastication is a complex physiological process aimed at breaking down food, mixing the resulting fragments with saliva and thus enabling swallowing. Masticatory performance or efficiency are usually evaluated by assessing the particle size distribution of natural or artificial test food, e.g. carrots or standardized silicone-based mixtures after a certain number of chewing strokes with the use of a sieve system [1–4]. As this method is elaborate and time-consuming, Liedberg et al. developed a test using two-coloured chewing gums [5]. This technique was developed further and validated using digital image processing to assess the degree of blending [6, 7]. However, it must be noted that this method can only evaluate mixing ability rather than comminution.

Although some studies indicate that impaired masticatory function does not necessarily lead to nutritional deficits, pointing to the fact that nutrition is multifaceted and influenced by various factors, such as personal habits and cultural influences [8–12], numerous studies have shown that it may lead to an inadequate food selection due to dietary avoidance patterns [13–19]. Moreover, a reduced chewing ability is also associated with a number of diseases, such as depression and dementia [20, 21]. Animal research indicates that reduced chewing activity in older animals can result in diminished learning abilities, neuro-endocrine alterations, and hippocampal degeneration [22–25]. Clinical studies also support a long-term link between mastication and cognitive function in older adults, suggesting a possible causal relationship, though this has not been definitively proven [26]. A recent study also suggests that high masticatory ability can help to alleviate stress [27]. 

Additionally, cross-sectional studies have consistently linked masticatory performance with frailty [28–30], which is positioned between health and the need for nursing care, and can often be reversed with early intervention. It increases mortality and diminishes physical abilities, underscoring the importance of early detection. Adequate nutrition, particularly sufficient energy and protein intake, is crucial for frailty prevention [31]. In 2019, a systematic review of longitudinal studies equally confirmed the association between masticatory function and the onset of frailty [32]. 

Many of the known influencing factors concern the dental status, mainly tooth loss and the presence of removable dentures [2, 5, 33, 34]. However, demographic characteristics may also play an important role. This includes the influence of sex, as men have a higher masticatory performance than women [35–37].

Regarding the potential impact of age, numerous age-related alterations in the oral system have been documented. These include a reduction in the size of masticatory muscles [38], a decline in electromyographic activity [39], a decrease in salivary flow rate [40], which in turn associates with reduced masticatory performance [41, 42], as well as changes to gustation and odor perception. However, a 2017 review by Peyron et al. suggests that despite such changes, ageing alone reportedly has little impact on an individual’s ability to grind foods [43]. . While bite force diminishes in older adults with compromised dental status, there appears to be little age-related difference when considering dentition [44, 45] In a population-based MRI study involving 747 individuals, age exerted a heterogeneous effect on masticatory muscles. For instance, the cross-sectional area of the lateral pterygoid muscle decreased with age among women but showed no age-dependence among men. The masseter muscle exhibited a weak association with age but demonstrated a strong correlation with the number of teeth in both genders [46] In a multivariate model that analyzed masticatory performance in 631 dentate subjects, age and sex did not significantly influence masticatory performance, either directly or indirectly through factors such as masseter cross-sectional area, temporomandibular disorders, and bite force. Instead, the number of functional tooth units and bite force emerged as the primary determinants of masticatory performance [47]. A concurrent review reports insights from a variety of animal and human studies which propose that ageing could potentially affect jaw sensorimotor functions. However, it equally highlights that the evidence regarding age-related alterations in voluntary control of jaw muscles remains ambiguous, largely due to confounding factors like sex, dentition, and inconsistencies across studies in terms of age ranges and tests employed [48]. It is thus frequently stated that age-related damage to the dentition accounts for most of the decline in masticatory performance, while age per se has little influence [47, 49, 50]. 

However, the age range of so-called “seniors” now spans many more decades, owing to advancements in health care, hygiene, reduced child mortality, adequate nutrition, and enhanced medical care, all of which contribute to increased life expectancies. Given the expectancy of today’s younger population to attain centenarian age [51], and with improvements in oral health, it is plausible to hypothesize that age-related changes to dentition may become visible and may significantly impact masticatory performance in the foreseeable future. To date, no study has systematically evaluated masticatory function in age groups extending up to 100 years or beyond.

Therefore, this pilot study aims to assess the potential impact of age on masticatory performance within age groups ranging from 65 to 106 years old, while accounting for a number of important confounders.

## Materials and methods

This study is part of the Heidelberg Dental Centenarian Study (HD-100Z), which is a cross-sectional survey and oral clinical examination among centenarians living in South-Western Germany. The study was approved by the local review board of the Medical Faculty of the University of Heidelberg (S-168/2019) and registered with the German Clinical Trials Register (DRKS 00017128, date of registration: 20/05/2019). In view of sample sizes reached by previous studies on centenarians [52–54], the recruitment target was set at 50 participants. Informed written consent was obtained from all study participants, as well as their legal guardians if applicable. This study followed the Strengthening the Reporting of Observational Studies in Epidemiology (STROBE) guidelines [55].

In total, 477 persons born before 1920 and registered in the catchment area (from Karlsruhe in the south to Darmstadt in the north and from the Rhine-Palatinate district in the east to the Neckar-Odenwald district in the west) were invited for study participation with no initial exclusion criteria; contact details were provided by the 183 registries in the area. Of the 477 persons identified, 55 individuals were deceased or no longer living under the registered address at the time of contact. Among 422 valid contacts, 117 refused participation and no contact could be established in 250 cases. Of 55 centenarians included in the study, 31 agreed to participate in the assessment of masticatory performance and were consequently included in this analysis. The centenarians were visited at their residences between May and October 2019. Participants for the control groups, aged either 65–74 years or 75–99 years old, were recruited from patients at the dental school of Heidelberg University Hospital between January 2020 and July 2022. Out of the 83 control patients aged between 65 and 99 years who were recruited and examined, the 62 (31 per group) who matched the centenarian group best were chosen based on sex, prosthetic status, and number of teeth using nearest neighbor matching. This process resulted in the categorization of three different age groups (*n* = 31 each) for the purpose of this analysis: Age group 1 (individuals aged 100 years and above), group 2 (those aged between 75 and 99 years), and group 3 (people aged between 65 and 74 years).

### Clinical investigation

Each participant underwent an oral examination, including their dental (e.g. number or teeth) and prosthetic status. The main type of prosthesis worn was categorized according to Kerschbaum [56] and then grouped as 1.“no removable denture/ fixed dental prosthesis”, 2.“removable partial denture” and 3.“complete denture” (referring to the weaker jaw, i.e. the jaw with the higher category of dental prosthesis). Moreover, the type of removable prosthesis was noted according to the 5th German Oral Health Study (DMS V) [57]: Resin partial denture, model casting denture, combined dentures (double-crown retained prosthesis), complete denture.

**Fig. 1:**
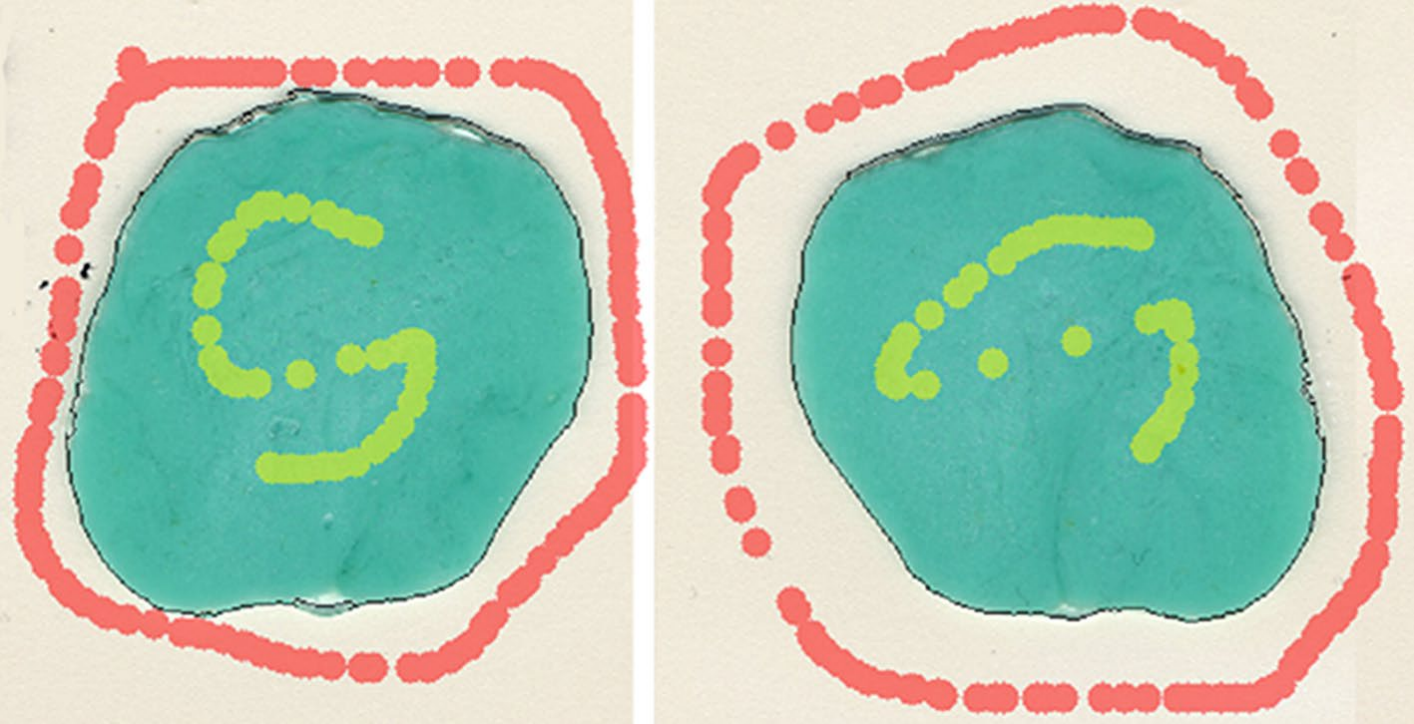
Exemplary analysis of a well-mixed bolus in the ViewGum© software, which identifies the gum outline (black line) after manual marking of the inside (yellow) and outside (red) surfaces

For the two-colored chewing gum test, specimens were prepared according to Schimmel et al. [58, 59] from Hubba-Bubba Tape Gums® in the flavors ‘Sour Berry® and ‘Fancy Fruit®. Strips of 30 mm length were cut and manually stuck together, so that the test strips were 30 × 18 × 3 mm. Specimens were inspected visually by 2 raters after 20 chewing cycles and subsequently scanned and assessed opto-electronically using the ViewGum© software, which measures the color of the pixels and computes the circular standard deviation of the hue component [59]. The higher the value, the more un-mixed the two colors are. Figure 1 shows the exemplary analysis of a well-mixed bolus in the software, which identifies the gum outline (black line) after manual marking of the inside (yellow) and outside (red) surfaces.

For further information regarding interview and clinical examination methods, dental health behaviors, caries experience, functional capacity, periodontal conditions, microbial parameters and oral health-related quality of life, please see our previous publications [60–63].

### Statistical analysis

Data analysis was performed with SPSS, Version 24.0 [64] and R, Version 4.2.2 [65]. Control groups were matched according to sex, prosthetic status and number of teeth using nearest neighbor matching. Characteristics of the study population were presented by means of descriptive statistics. For continuous variables mean ± SD, median (Q1-Q3), minimum and maximum were reported, whereas for categorical variables absolute and relative frequencies were reported. The age groups were compared using the Fisher’s exact test for categorical variables and the Kruskal-Wallis’s one-way ANOVA for continuous variables. All statistical tests were two-sided and conducted at the 5% significance level. Further, a multiple linear regression analysis was performed to assess the effect of patients’ age, sex, number of teeth, type of prosthesis on the standard deviation of hue. The estimated effects alongside 95% confidence intervals and p-values were reported. Prior to model fitting, we assessed multicollinearity among these predictors using the Variance Inflation Factor (VIF). The assumption of independent residuals was verified using the Durbin-Watson test. We employed the Shapiro-Wilk test and a Q-Q plot to check the normality of residuals. Homoscedasticity was assessed via visual inspection of a plot of residuals against fitted values. We conducted a visual inspection of scatter plots for each variable against the dependent variable (standard deviation of hue) to identify any potential outliers and to verify the linearity assumption required for multiple linear regression (Appendix Tables A.2–A.4, Figures A.1–A.3). Due to the nature of the study, no formal sample size calculation was performed and p-values are to be interpreted in a descriptive sense.

## Results

Characteristics of the study populations are summarized in Table 1. The age distribution across the three study groups was as follows: age group 1 had a mean age of 101.1 years (median = 101 years), age group 2 had a mean age of 81.0 years (median = 80 years), and age group 3 had a mean age of 70.3 years (median = 70 years). Most were female (77%). The majority of participants had a complete denture (60%), followed by a removable partial denture (31%) and no removable denture or fixed dental prosthesis (9%). There were no significant differences regarding sex, type of prosthesis or number of teeth between the three age groups.

**Table 1:** Characteristics of the study populations

	Age group 1 100 + yrs *n* = 31	Age group 2 75–99 yrs *n* = 31	Age group 3 65–74 yrs *n* = 31	Total *n* = 93	*p*
**Sex**					
Male	5 (16%)	11 (35%)	5 (16%)	21 (23%)	0.152^Fish^
Female	26 (84%)	20 (65%)	26 (84%)	72 (77%)	
**Main type of prosthesis (referring to the weaker jaw)**					
No removable denture/ fixed dental prosthesis	3 (10%)	1 (3%)	4 (13%)	8 (9%)	0.260^Fish^
Removable partial denture	6 (19%)	11 (35%)	12 (39%)	29 (31%)	
Complete denture	22 (71%)	19 (61%)	15 (48%)	56 (60%)	
**Type of prosthesis maxilla**					
No removable denture/ fixed dental prosthesis	3 (10%)	2 (6%)	4 (13%)	9 (10%)	0.295^Fish^
Removable partial denture	7 (23%)	11 (35%)	14 (45%)	32 (34%)	
Complete denture	21 (68%)	18 (58%)	13 (42%)	52 (56%)	
**Type of prosthesis mandible**					
No removable denture/ fixed dental prosthesis	10 (32%)	10 (32%)	10 (32%)	30 (32%)	0.998^Fish^
Removable partial denture	10 (32%)	11 (35%)	12 (39%)	33 (35%)	
Complete denture	11 (35%)	10 (32%)	9 (29%)	30 (32%)	
**Number of teeth**					
Mean ± SD	5.3 ± 6.3	7.6 ± 6.8	9.0 ± 8.2	7.3 ± 7.2	0.192^KW^
Median (Q1-Q3)	2 (0–10)	7 (0–13)	8 (0–17)	6 (0–13)	
Min-Max	0–22	0–21	0–26	0–26	
**Total number of occlusal pairs (incl. fixed and removable dental prosthesis)**					
Mean ± SD	12.2 ± 2.9	11.8 ± 2.5	12.2 ± 1.9	12.1 ± 2.5	0.203 ^KW^
Median (Q1-Q3)	14 (12–14)	12 (10–14)	12 (12–14)	12 (12–14)	
Min-Max	5–14	4–14	5–14	4–14	
**Number of occlusal pairs: Natural teeth and fixed dental prosthesis**					
Mean ± SD	1.4 ± 3.1	1.2 ± 3.0	1.9 ± 3.7	1.5 ± 3.2	0.495^KW^
Median (Q1-Q3)	0 (0–0)	0 (0–0)	0 (0–3)	0 (0–0)	
Min-Max	0–12	0–12	0–13	0–13	
**Number of occlusal pairs: Removable dental prosthesis**					
Mean ± SD	8.4 ± 6.4	7.5 ± 6.4	7.7 ± 5.7	7.9 ± 6.1	0.463^KW^
Median (Q1-Q3)	12 (0–14)	12 (0–14)	11 (0–12)	12 (0–14)	
Min-Max	0–14	0–14	0–14	0–14	
**Number of occlusal pairs: Mixed**					
Mean ± SD	2.5 ± 3.7	3.1 ± 4.2	2.6 ± 3.8	2.7 ± 3.9	0.815^KW^
Median (Q1-Q3)	0 (0–5)	0 (0–6)	0 (0–5)	0 (0–5)	
Min-Max	0–11	0–12	0–13	0–13	

^Fish^ Fisher’s exact test

^KW^ Kruskal-Wallis’s one-way ANOVA

### Masticatory performance in the three age groups

**Fig. 2:**
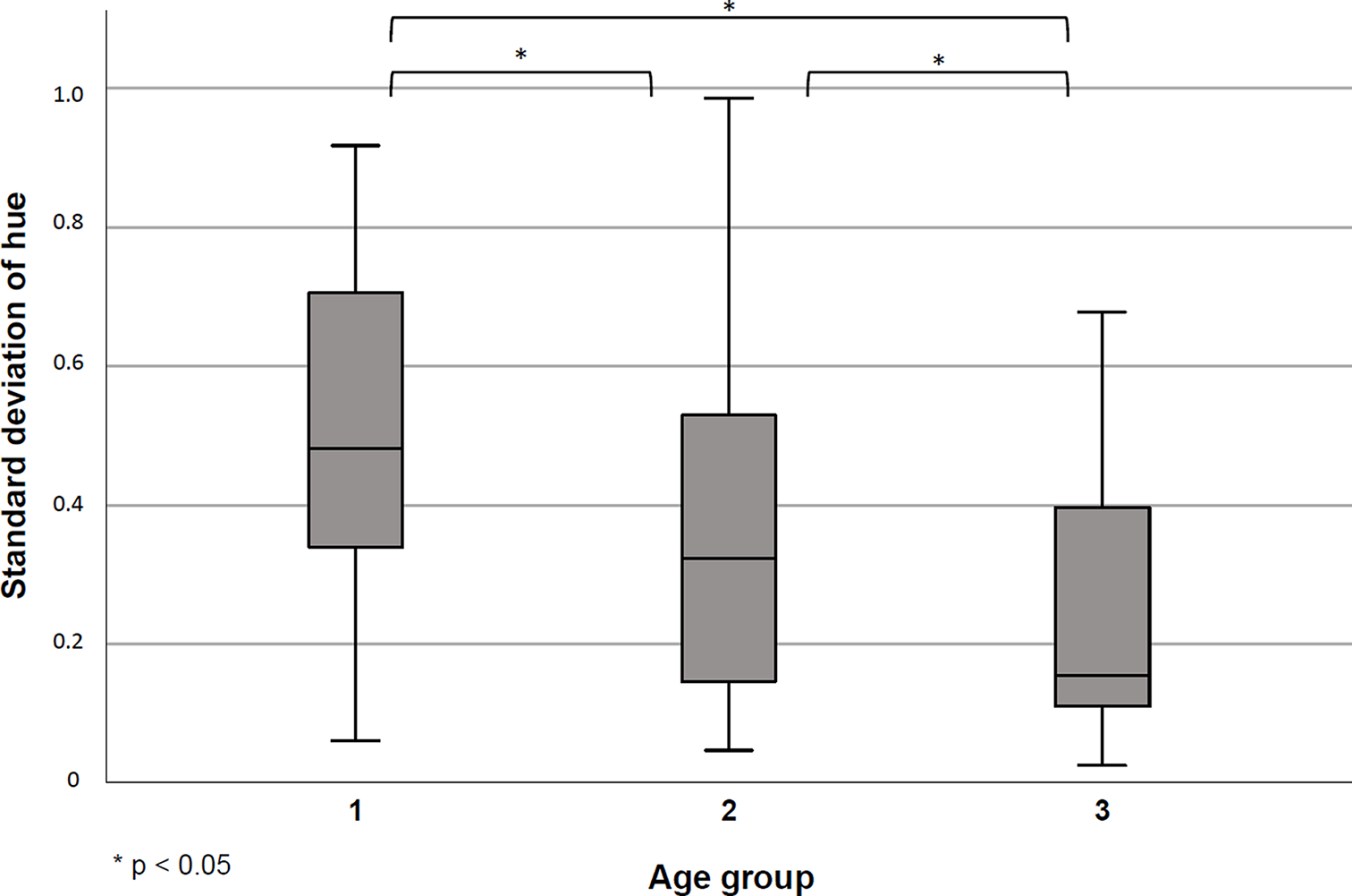
Standard deviation of the hue value between the three age groups. (1 = 100–106 years; 2 = 75–97 years, 3 = 65–74 years)

The standard deviation of hue differed between the three groups (*p* < 0.05), the mean was 0.51 ± 0.24 (min-max: 0.06–0.92) for the centenarian group, 0.36 ± 0.245(min-max: 0.05–0.98) for individuals aged 75–99 years old, and 0.24 ± 0.19 (min-max: 0.03–0.68) for those aged 65–74 years old. These findings indicate that the chewing gum was more effectively mixed in the younger age groups (Fig. 2).

The multiple linear regression model (Table 2), including patients’ age (categorized), sex, number of teeth and type of prosthesis (referring to the weaker jaw) as independent variables showed that the masticatory performance decreased significantly between the centenarian group and both age group 2 (-0.11, *p* = 0.037) and age group 3 (-0.21, *p* < 0.001). The decrease being more pronounced when compared to individuals aged 65–74 (age group 3) as opposed to individual aged 75–99 (age group 2) suggests that the standard deviation of hue tends to be higher with age, i.e. the masticatory performance decreases. Sex, number of teeth, and type of prosthesis did not show statistically significant effect on the hue deviation.

To validate these findings and to assess the impact of treating age as a continuous variable, a supplementary regression analysis was conducted with age as a numerical factor. This analysis, detailed in Appendix Table A.1, corroborates the results obtained from the categorical model.

**Table 2:** Results of the multiple linear regression model predicting the standard deviation of hue

Variable	Estimate	95% CI			
		Lower bound	Upper bound	p-value	
Age in years: 100+ (reference)	0				
Age in years: 75–99	-0.112	-0.217	-0.007	**0.037**	
Age in years: 65–74	-0.207	-0.311	-0.103	**<0.001**	
Sex: male (reference)	0				
Sex, female	0.001	-0.102	0.104	0.988	
Number of teeth	-0.007	-0.016	0.002	0.135	
Type of prosthesis: no removable denture/ fixed dental prosthesis (reference)	0				
Type of prosthesis: removable partial denture	-0.063	-0.237	0.111	0.473	
Type of prosthesis: complete denture	0.101	-0.107	0.308	0.337	

## Discussion

The present study aimed to assess the influence of age on masticatory performance in age groups ranging from 65 to 106 years old. The findings revealed that the mean standard deviation of hue, a measure of masticatory performance, varied across the age groups, indicating a decline in masticatory performance with increasing age. In practical terms, the differences in the standard deviation of hue values observed among the age groups are clinically perceivable and are akin to those observed between an edentulous person using removable dentures and a dentate person [6, 59]. Previous studies have shown that differences in hue values, ranging between 0.1 and 0.2 (similar to the differences between our groups), correspond to noticeable changes in masticatory performance, typically from category 3 (‘bolus slightly mixed’) to category 4 (‘bolus well mixed’) in visual assessments of the bolus [7, 59]. 

It has been commonly suggested that age-related changes in the dentition contribute to the decline in masticatory performance, while age itself has little influence [47, 49, 50]. However, within the limitations of this study, our results indicate that age may have a significant effect masticatory performance. The age group commonly categorized as “old” has become increasingly diverse in the last decades. In contemporary societies, advancements in health management have given rise to two distinct age categories: the “young old”, who are very similar to the typical adult age group, and the “very old” who experience rapid health deterioration and frailty. Therefore, effects that are not so apparent in ‘younger’ age groups may become evident as people age further. It is commonly known that muscle mass decreases with age [66–68]. The atrophy of muscle fibers, particularly the fast-contracting fibers, leads to muscles that are more fatigable, weaker, and slower [69]. The loss of muscle fibers is accompanied by a decrease in muscle innervation and a reduced number of functional motor units [70]. However, the correlation between muscle mass and its functional effectiveness is not always straightforward, as many systems have the ability to adapt [71, 72]. Initially, adaptive changes such as sprouting of motor terminals and re-innervation of neighboring muscle fibers can compensate by producing greater force, albeit less efficiently and with less coordinated muscle contractions [73]. .Additionally, age-related skeletal muscle atrophy appears to be a muscle group-specific process [74], and training can help prevent it. The masticatory muscles are among the few muscle groups that remain comparably active even in old age. This could explain why the effect of age may have been overshadowed by factors such as dental status.

It is thus probable that while impaired chewing ability may not be strongly associated with age in the healthy “young old” population, it is more likely to be observed in the “very old” group [43], where the age-related decline is more pronounced and the adaptation mechanisms are exhausted. With further aging, the capacity for axonal sprouting and re-innervation decreases, leading to an increase in denervated myofibers which undergo disuse atrophy or are replaced with fat and fibrous tissue, further contributing to the decline in muscle mass, strength, and power [48]. Certainly, the number of natural teeth is important for chewing function. Yet, based on this study, there appears to be a notable trend within this population suggesting that extreme old age may play a substantial role. Emphasizing the unique aspect of our study population is therefore of importance. To our knowledge, no previous study has assessed masticatory function in age groups reaching up to 100 years or more. The inclusion of centenarians in our study provides valuable insights into masticatory performance in this specific age group, which has received limited attention to date.

Moreover, our study explored the influence of various demographic factors on masticatory performance. While age emerged as a potential significant influencing factor, other factors such as sex, number of teeth, and type of prosthesis did not show statistically significant effect on masticatory performance.

It is important to acknowledge some limitations of our study. Firstly, the sample size (31 participants per group) was relatively small and no formal sample size calculation was performed, which may limit the generalizability of our findings. Future studies with larger sample sizes are warranted to validate and expand upon our findings. Although we considered sex, type of prosthesis or number of teeth as some of the most important confounders, there are, there are additional factors that could influence masticatory performance, which we could not also consider due to feasibility reasons. This includes dementia or other physical diseases, as well as maximum bite force, salivary function, or the coordination of movements involving the jaw, tongue, cheeks, and lips [75]. These functions, which deteriorate with age [76], might have impacted our findings, suggesting that the observed decline in masticatory performance with age could also be related to these unmeasured factors. Additionally, our study focused on older adults ranging from 65 to over 100 years of age. While this approach provided valuable insights into age-related changes in masticatory performance among older adults, it also posed certain limitations regarding the broader applicability of our findings. However, recruitment challenges in our region made it difficult to find younger individuals with a level of prosthetic dentistry similar to that of our centenarian participants. These recruitment difficulties reflect broader demographic trends, such as improved dental health in younger populations, which limit the availability of younger participants who meet the specific criteria required for a perfectly matched study. Future studies could potentially expand the scope of investigation to include younger age groups with comparable prosthetic needs, should recruitment possibilities improve.

Moreover, our study focused solely on the standard deviation of hue as a measure of masticatory performance. Further investigations utilizing additional objective measures, such as particle size distribution analysis, could provide a more comprehensive understanding of masticatory performance.

In conclusion, our study contributes to the existing literature by examining masticatory performance in age groups ranging from 65 to 106 years old, including centenarians. The findings highlight a potential decline in masticatory performance with increasing age, contradicting the notion that age alone has minimal impact on masticatory ability. These findings underscore the need for interventions and support in maintaining adequate masticatory function in the older population. Further research with larger sample sizes and comprehensive assessment methods is necessary to corroborate these findings and guide clinical practice in optimizing masticatory performance in elders.

### Electronic supplementary material

Below is the link to the electronic supplementary material.

Supplementary Material 1

Supplementary Material 2

## Data Availability

Data is available from the corresponding author upon reasonable request.
