# Supplementary material for: Very old age impacts masticatory performance: a study among sexagenarians to centenarians
Source: Clin Oral Investig. 2024 Jun 1;28(6):349. doi: 10.1007/s00784-024-05742-y (PMC11144126; doi:10.1007/s00784-024-05742-y)
Supplement: Supplementary file 2 — Supplementary Material 2 [file 784_2024_5742_MOESM2_ESM.docx]

**Appendix**

| **Table A.1** Results of the multiple linear regression model predicting the standard deviation of hue (age as a numerical variable) | | | | |  |
| --- | --- | --- | --- | --- | --- |
| **variable** | **Estimate** | **95% CI** | |  |  |
|  |  | **Lower bound** | **Upper bound** | **p-value** | |
| Age in years | 0.006 | 0.002 | 0.009 | **<0.001** | |
| Sex: male (reference) | 0 |  |  |  | |
| Sex, female | -0.016 | -0.118 | 0.087 | 0.763 | |
| Number of teeth | -0.007 | -0.016 | 0.002 | 0.148 | |
| Type of prosthesis: no removable denture/ fixed dental prosthesis (reference) | 0 |  |  |  | |
| Type of prosthesis: removable partial denture | -0.058 | -0.233 | 0.117 | 0.514 | |
| Type of prosthesis: complete denture | 0.111 | -0.097 | 0.32 | 0.291 | |
|  | | | | |  |

**Table A.2:** Variance Inflation Factor (VIF) for each predictor used in the multiple regression analysis to assess the level of multicollinearity

| **Variable** | **VIF** |
| --- | --- |
| Age group | 6.67 |
| Gender | 4.10 |
| Type of prosthesis | 6.38 |
| Number of teeth | 2.31 |

**Table A.3:** Durbin-Watson Test for Residual Independence

| **Description** | **Value** |
| --- | --- |
| Durbin-Watson Statistic | 2.09 |

**Table A.4:** Shapiro-Wilk Test for Normality of Residuals

| **Description** | **Result** | **P-value** |
| --- | --- | --- |
| Shapiro-Wilk Statistic | 0.975 | 0.073 |


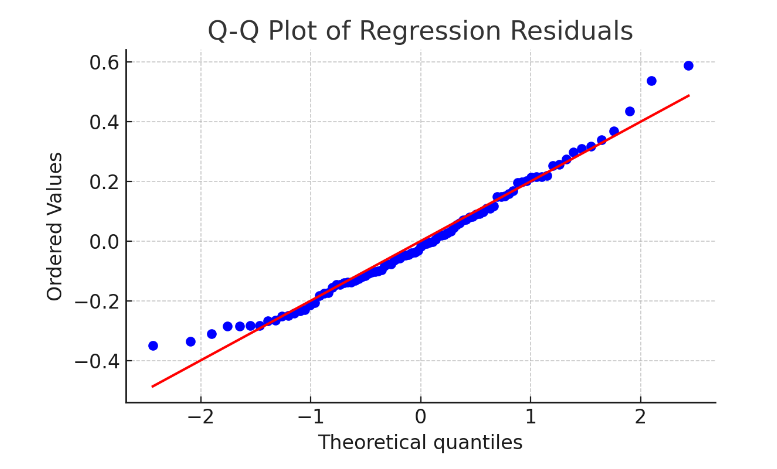


**Figure A.1 Quantile-Quantile (Q-Q) Plot of Regression Residuals**

**
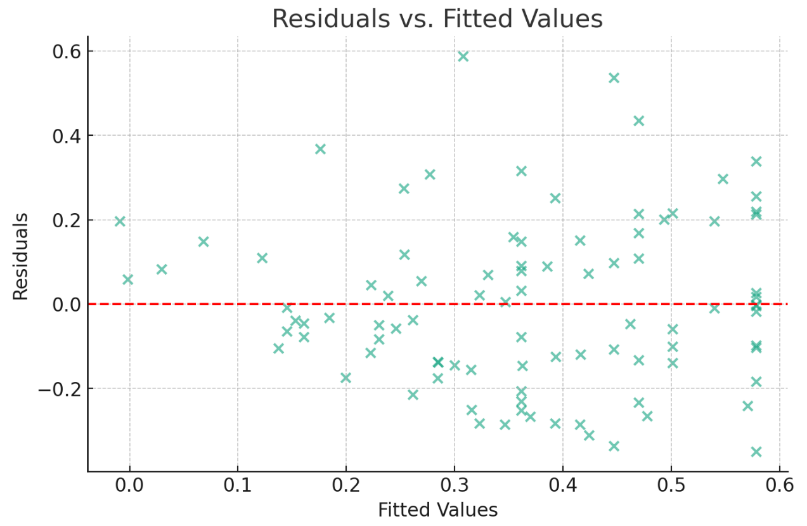
**

**Figure A.2 Plot of Residuals versus Fitted Values to Assess Homoscedasticity**

**
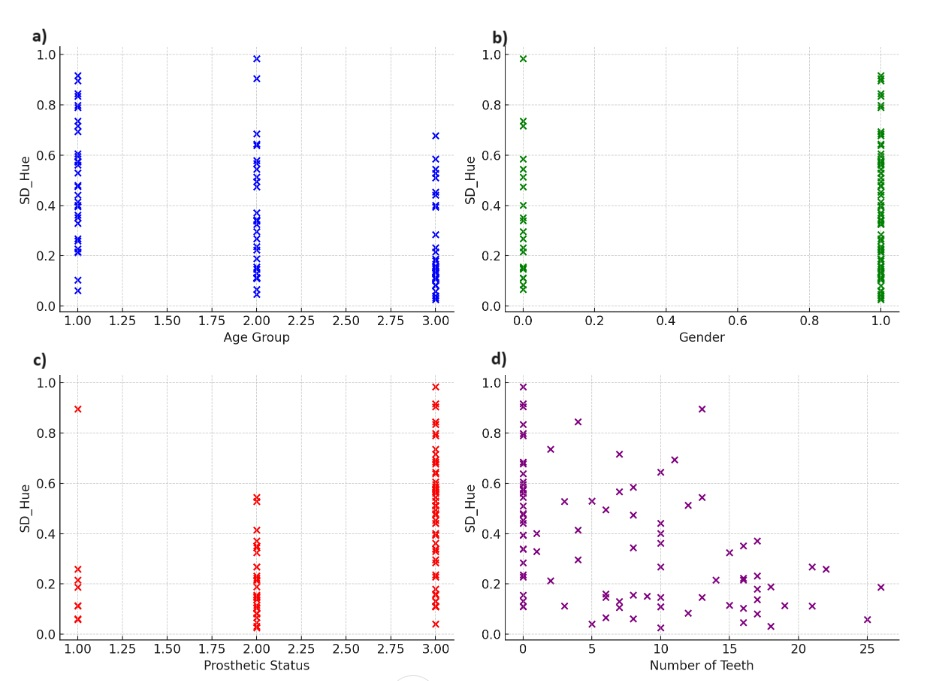
**

**Figure A.3 Scatter Plots of Predictors vs. Masticatory Performance (Standard deviation of Hue, SD Hue) a) Age group (1 = ≥100; 2 = 75-99; 3 = 65-74), b) Gender (0 = male; 1 = female), c) Prosthetic status (1 = natural teeth/fixed dental prosthesis; 2 = partial prosthesis; 3 = complete prosthesis), d) Number of teeth**
